# The Body I Live in. Perceptions and Meanings of Body Dissatisfaction in Young Transgender Adults: A Qualitative Study

**DOI:** 10.3390/jcm9113733

**Published:** 2020-11-20

**Authors:** Marta Mirabella, Guido Giovanardi, Alexandro Fortunato, Giulia Senofonte, Francesco Lombardo, Vittorio Lingiardi, Anna Maria Speranza

**Affiliations:** 1 Department of Dynamic and Clinical Psychology, Sapienza University of Rome, Via degli Apuli 1, 00185 Roma, Italy; guido.giovanardi@uniroma1.it (G.G.); alexandro.fortunato@uniroma1.it (A.F.); vittorio.lingiardi@uniroma1.it (V.L.); annamaria.speranza@uniroma1.it (A.M.S.); 2 Laboratory of Seminology, Sperm Bank “Loredana Gandini,” Department of Experimental Medicine, Sapienza University of Rome, 00185 Roma, Italy; giulia.senofonte@uniroma1.it (G.S.); francesco.lombardo@uniroma1.it (F.L.)

**Keywords:** gender incongruence, body dissatisfaction, body image, appearance, negative outcomes, eating disorders, sexuality

## Abstract

Body dissatisfaction in individuals with Gender Incongruence (GI) represents a primary source of suffering. Several studies have highlighted how this suffering has psychological, physical, and biological implications. This work aims to explore experiences related to body dissatisfaction and investigate the issues associated with living in a body perceived as incongruent for individuals with GI. Thirty-six individuals, aged between 18 and 30 years old and at stage T0 of hormone treatment, participated in the study. Body dissatisfaction and experiences related to it were investigated using the Clinical Diagnostic Interview. The Consensual Qualitative Research methodology was applied to the transcripts of the interviews. Several themes emerged: experiences with GI development, experiences with puberty and bodily changes, perception of one’s body, psychological problems and complex behavioral patterns related to body dissatisfaction. Results pointed out the complexity implied in the relationship with one’s body for individuals with GI, highlighting specific aspects of body dissatisfaction among these individuals (e.g., eating disorders, sexual difficulties, social withdrawal). This study underlines the need for a deeper understanding of some aspects of GI to better define guidelines for a correct assessment of it. In this way it will be easier to avoid negative outcomes for the psychological and general health of transgender people.

## 1. Introduction

Body dissatisfaction consists of a negative evaluation of one’s appearance, and it is considered the core aspect of uneasiness in individuals with gender dysphoria (GD) [1]. The Diagnostic and Statistical Manual of Mental Disorders (DSM) [2] defines GD as a marked incongruence between the individual’s perceived gender identity and the gender assigned at birth. Such incongruence is also associated with clinically significant distress or impairment in important areas of functioning [2]. This definition was adopted in the last version of the DSM in order to reduce stigma, likewise securing access to care. Recently, the International Classification of Diseases 11th Revision (ICD-11) [3] proposed a different definition of this condition, introducing the term “gender incongruence (GI)” instead of GD, no longer classifying it as a mental disorder but as a condition related to sexual health [3]. This article will use the term “gender incongruence” to define individuals who perceive a discrepancy between their assigned gender at birth and their gender identity. So far, several studies explored body perception and body dissatisfaction in individuals with GI [1,4,5,6], considering it as not only related to secondary sexual characteristics but also associated with non-sexual body features, body shape, and physical appearance. These studies underlined how such discomfort could lead GI individuals to engage in maladaptive behaviors, such as self-harm or restrictive/uncontrolled eating, in order to alter their body [7]. Thus, in individuals with GI, body dissatisfaction represents a potential risk factor for developing psychopathologies and body image disturbances. Specifically, two different population studies [8,9] conducted among individuals with GI, cisgender people whose sex assigned at birth is aligned with their gender identity [10] and individuals with disordered eating behaviors, pointed out that individuals with GI show higher rates of disordered eating patterns and body dissatisfaction than cisgender people, but less than individuals with eating disorders. Some case report studies have also discussed disordered eating behaviors in adults [11,12,13,14,15] and youths [16,17,18,19,20] with GI, ascribing body dissatisfaction related to GI as one of the core aspects of the co-occurrence between these two conditions. Such studies considered eating disorder behaviors (e.g., food avoidance and extreme weight loss) as symptoms of inner conflict and a dysfunctional coping strategy adopted to avoid unwanted pubertal changes. In this regard, several studies (see two recent reviews [21,22]) tend to support the evidence that hormone therapy reduces body dissatisfaction, social distress and low self-esteem, improving GI individuals’ overall well-being at different levels. More specifically, regarding eating disorders and body dissatisfaction in individuals with GI, some studies [7,23] also pointed out differences among assigned female at birth (AFAB) trans people and assigned male at birth (AMAB) trans people. Indeed, AFAB individuals described restrictive eating patterns related to the will to suppress their body shapes, while AMAB individuals showed major body checking and weight concerns related to the desire to emphasize their femininity. Moreover, research addressing self-harming in two different samples (one taken from the general population and the other referred to a gender clinic) of gender non-conforming individuals has shown that body discomfort and weight concerns represent risk factors for self-harm and suicide attempts in transgender individuals [24,25]. Considering its various levels of analysis, body discomfort in individuals with GI is a factor that needs more in-depth understanding. So far, the difficulty of perceiving a satisfying body image for individuals with GI has been assessed mostly through self-report tools [1,26,27,28,29]. However, some other studies [30,31,32] have pointed out the need to consider this phenomenon from a qualitative perspective as well.

Therefore, the present work aims to investigate through a qualitative study the personal meaning that individuals with GI give to body dissatisfaction, in order to better understand its potential role as a risk factor for psychopathological outcomes.

## 2. Methods

### 2.1. Participants

The sample comprised 36 participants, 15 AMAB and 21 AFAB, aged 16–30 (M = 21.11). All participants were at stage T0 of hormonal therapy (waiting to begin it) at the Unit of Endocrinology of the Policlinico Umberto I Hospital of Rome and defined themselves as transgender. The inclusion criteria were being at stage T0 of hormonal therapy and not older than 30 years old (see Table 1).

### 2.2. Measures

The Clinical Diagnostic Interview (CDI) [33] was used, and its transcripts analyzed. CDI is a 2-to-3-h-long interview used for clinical and research purposes. It consists of 14 questions investigating the respondent’s personal history, their affects, relationships, behaviors, affective states, emotion regulation processes, and cognitive patterns [33]. Clinicians ask respondents to tell them about their lives, and then they systematically evaluate the interviewees’ ways of thinking, feeling, regulating emotions, and self or other representations [34]. The questions order and formulation should be adapted based on the interviewer’s clinical skills, and on the themes that emerge during the interview. Additionally, literature shows that CDI allows for collecting personal psychological contents (experiences, thoughts, wishes, fantasies...) clinically significant, comparable to those provided by the Structured Clinical Interview (SCID), thus being a useful tool for clinician and researchers [35].

### 2.3. Procedure

The study was conducted by trained researchers at the Policlinico Umberto I hospital of Rome, in the Unit of Endocrinology. Participants were asked to provide written informed consent; if they were minors, consent was given by parents. Participants were reminded that their responses would be confidential and that their involvement in the study could be terminated at any time. The study was carried out with the approval of the Ethics Committee of the Department of Developmental and Clinical Psychology, Sapienza University of Rome.

All interviews were audiotaped and transcribed verbatim, removing any information that could identify the participants. The interview transcripts were then analyzed using the Consensual Qualitative Research methodology [36]. This rigorous and systematic qualitative approach aims to explore interviewees’ subjective experiences through the work of a group of researchers. Researchers who use this approach have to identify general themes (domains) present in the interviews and extract specific core ideas for each domain. Core ideas capture the essence of each domain in fewer words, given a representative example [37]. Moreover, researchers have to compare the core ideas in order to arrive at a consensus list of core ideas for each domain. Each researcher then independently examines the core ideas of each domain trying to identify common elements and then cluster together similar core ideas into categories. Following this process, the team discusses together the preliminary categories each member found, until a consensus is reached about what category best represents specific themes across all data. Finally, an analysis of the frequencies to evaluate how the individual categories (and sub-categories) were representative of the entire sample is done. Researchers count cases that fall into one category and use conventional frequency denominations [38]: categories are labeled general when the associated core ideas are found in all, or in all but one or two, of the participants (8–10); typical when the core ideas are found in half or more than half of the participants (5–7); and variant when the associated core ideas are found at least in two participants and up to half of them (2–4) [37].

## 3. Results

Transcript analysis performed using the Consensual Qualitative Research (CQR) methodology [36] revealed four domains that can be divided into three different areas. The first two domains are related to body dissatisfaction and lack of self-acceptance and are both included in the area defined as ‘gender identity: self-perception’. The third domain is related to disordered eating patterns and is comprised in the area related to ‘eating behaviors’. The fourth domain is related to experiences related to sexuality and is included in the area defined as ‘sexuality’.

### 3.1. Gender Identity: Self-Perception

Transcript analysis revealed experiences related to the respondents’ perception of their gender identity and body (see Table 2).

As mentioned above, this area comprises two domains: body dissatisfaction and lack of self-acceptance. Regarding body dissatisfaction, individuals reported a growing sense of discomfort towards their appearance, with feelings of non-conformity starting from an early age. Within this domain, three categories emerged: discomfort, difficult experiences related to puberty, and self-harming/suicidal tendencies.

Regarding discomfort, which was present in almost every interview, the most frequent feelings reported by the participants’ were shame and repulsion towards their body, since childhood. All participants described situations in which they felt ashamed of and repulsed by their body and physical appearance.

“As a child, I knew that there was something wrong with me. I felt like I didn’t feel myself as I should have, and it made me sad. I remember telling my babysitter that I felt that I was someone else.”

(AMAB)

Other respondents reported having felt ashamed of their body image when growing up and thus adopting strategies to face those feelings, such as trying to conceal their body and gender.

“I felt inadequate, wrong. I felt like an outcast. I felt ugly, I felt clumsy... I didn’t want to show my body, I only wore oversized clothes... I couldn’t stand the thought of my body as it was.”

(AFAB)

Furthermore, with the onset of puberty, several difficult experiences associated with bodily changes emerged among most respondents. Specifically, they referred that childhood represented a “safe place” since it meant inhabiting a body in which gender differences were not yet evident, but that with puberty and the development of secondary sexual characteristics their feelings of uneasiness significantly increased, starting to be directly related to their body and physical appearance. This result is consistent with the literature [27], which underlines how the development of marked gender characteristics associated with puberty can lead individuals with GI to experience higher rates of discomfort and body dissatisfaction.

“When puberty arrived, it was horrible… everything was fine before. I was a male in my mind, I was a happy boy, but then everything changed. I tried to suppress it because I didn’t know how to deal with my body becoming something, I was not… so I started having panic attacks and feeling depressed.”

(AFAB)

“Puberty has been really tough. Growing up and realizing that there was something wrong with how I felt was really tough. All my teenage years were really tough. I started using drugs, drinking alcohol… I wanted to, like, stop feeling what I was feeling.”

(AMAB)

Moreover, some respondents reported self-harming behaviors, suicidal thoughts, and suicide attempts related to their body dissatisfaction, consistent with the literature [39,40]. Most of the respondents reported self-harming and suicidal thoughts, whereas accounts of suicide attempts were less frequent. All these experiences were described as present to different extents during various stages of the respondents’ life, with an increase associated with the onset of puberty and the development of gendered marked characteristics. Our results corroborate those of a study conducted by Skagerberg and colleagues [41], which pointed out how suicide attempts and self-harming behaviors are generally more common among adolescents and young adults than among older adults or prepubertal children.

Indeed, some respondents stated that they developed suicidal thoughts because they felt that their body was evolving in a different way than the one they desired; they felt unable to tolerate such changes and hopeless about the future. Regarding self-harm behaviors, respondents reported having cut, burned, and hit themselves in an attempt to feel something and canalize their suffering, in line with the literature [42].

“I used to burn my arms with a lighter... then I started to cut myself, first a bit and then a lot... and it made me feel better... I finally felt something real.”

(AMAB)

Self-harm seemed to be more common among AFABs (38% among the AFAB group) than among AMABs (20% among the AMAB group). Similar results were found concerning suicidal thoughts, which were more frequent among AFABs (50% among the AFAB group) than among AMABs (20% among the AMAB group). Most AFAB respondents reported having had—or still having—self-harming behaviors and/or suicidal thoughts. In contrast, in the AMAB group only a few individuals described self-harming behaviors and suicidal thoughts. These results seem in line with a study of Peterson and colleagues [43], which has shown that suicidal thoughts and self-harming behaviors are more common among AFABs than among AMABs.

“I never tried to kill myself, but I sure thought about it. I used to go online to search what the chances of success for each “method” were… so as to pick the best one, you know. But then I never did it. However, just knowing that I had the whole thing planned made me feel better. Besides, I hurt myself… I cut myself... and I told people they were scratches from playing with my cat. Thoughts like ‘I have to disinfect my cuts’ or ‘I have to do this’ satisfied me… they occupied a lot of space in my head… I had no time to think about other things, and it made me feel better.”

(AFAB)

“I had these thoughts about killing myself and ending my suffering, but I didn’t do it... I was a coward.”

(AFAB)

“When I was in middle school I used to cut myself to get attention. It was the only way to bond with others, the only thing that offered me some relief.”

(AFAB)

“No... I never attempted suicide or tried to harm myself. I’ve thought about it, yes. More frequently during adolescence, like when I was fifteen, sixteen years old–at that time I was constantly thinking about it, actually.”

(AMAB)

Only a few respondents reported having attempted suicide. No difference between AFABs and AMABs was found. However, many respondents reported not being able to accept themselves or their body.

“I felt like I couldn’t achieve what was expected from me… I felt like there was something wrong with me... so I took sleeping pills and other pills that I found in my house and woke up in the hospital...”

(AFAB)

“I did... I tried to do it. I even tried to do it when I was really young, secretly. But the moment it got really bad was when I was growing up–during my teenage years, I mean. In that period, I tried to choke myself to death, I tried to poison myself, I’d hit my head onto the wall hoping that my skull would shatter. I wanted to jump out of a window. I wanted to die.”

(AMAB)

Regarding the *lack of self-acceptance* domain, two categories emerged: perception of one’s diversity and social relations. Regarding the perception of one’s diversity, a feeling of non-conformity among others (often since childhood) was present among all individuals, as shown in Table 2. More specifically, respondents described a feeling of mismatch between their physical body and their perceptions from an early age, as well as a feeling of being different from their peers. Thus, respondents highlighted behaviors related to a desire to “change who they were”, for example, wearing clothes or playing with toys traditionally associated with the other gender or wanting to have long/short hair.

“I remember that, when I was in kindergarten, I used to wear these colorful skirts. We had a dresser full of princesses’ dresses and costumes in our classroom, and I liked to wear the most colorful ones. Thank god no one did anything to make me take them off. I felt so comfortable wearing those clothes.”

(AMAB)

Regarding the social relationships category, respondents stated that, during school age, a *lack of self-acceptance* started to significantly influence their daily lives, social relationships, and interactions. Indeed, as shown in Table 2, shame seemed to be the most frequent emotion felt by respondents during social situations. Many of them also reported having experienced physical and psychological bullying, whereas fewer respondents reported social withdrawal tendencies. Histories of failures at exams, school suspensions or expulsions were also reported. In this regard, a study by Pampati and colleagues [44] highlighted that, compared to cisgender youths, transgender individuals are significantly more likely to report bullying victimization.

“I had to change school two times because I was being bullied constantly. And then one day I simply stopped going. I just couldn’t handle it. I stayed home for several months without ever going out.”

(AFAB)

“I do not have any good memories about high school, because unfortunately, though I had long hair, I still looked like a boy, and I got teased all the time.”

(AMAB)

### 3.2. Eating Behaviors

From the analysis of transcripts, various experiences of disordered eating patterns emerged (see Table 3).

Within this domain, two categories emerged: one related to a disordered eating history and one concerning individuals’ awareness of its underlying causes. Regarding the first category, respondents described past and present experiences of restrictive and bulimic behaviors, binge eating, weight and shape concerns, and compulsive body-checking. In line with the literature [4,11,14,45], these behaviors appeared related to a severe body dissatisfaction and an internal sense of discomfort with one’s body. However, transcript analysis also highlighted how individuals were aware of the underlying causes of their disordered eating behaviors, as the second category shows. Specifically, respondents reported having consciously engaged in disordered eating conducts. They highlighted how their motivations were related to a desire to alter their appearance in order to align their physical body to their perceived and desired gender.

Restrictive conducts emerged as the most common among respondents, which often reported behaviors such as skipping meals, restricting, counting calories, and developing excessive attention to their body and weight. Restrictive conducts seemed to be more common among AMAB respondents (60% of the AMAB group) than among AFAB respondents (23% of the AFAB group). Indeed, among AMAB respondents emerged a desire to lose weight and to exert strict control over their body to look more slender. Among AFAB respondents, restrictive conducts were less frequent, even though still characterized by excessive attention toward body appearance and a severe desire to control caloric/food intake.

Regarding the underlying causes of these behaviors, respondents described being driven by a desire to alter their gender marked characteristics and attack or exert control over their body. Some reported being driven by a wish to look more slender and more in line with their desired gender, and they admitted to having skipped meals or avoided social situations that involved eating. Other respondents reported having engaged in severe caloric restriction, which resulted in a significant loss of weight because of their feelings of distress, low self-esteem, and a desire to damage and self-harm themselves.

“It started with me wanting to be slimmer, but then I realized that it was about something else. Losing weight meant having flatter breasts and slimmer hips. It meant looking more masculine.”

(AFAB)

“It’s been a year since I’ve started to be a little obsessed with dieting and calories. I think about calories all the time. I think about food, about having to restrict, about what food will do to my body if I eat it. I’m eating pasta and I start to think about what would happen if I eat some more, for example. But I do not want this thing to become something serious. I just want to get to a certain weight, that is 60 kg. I just want to look a little bit more slender and feminine.”

(AMAB)

“Let’s just say that I had lost a lot of weight. I used to weight 85 kilos. At first, I simply started following a diet avoiding pasta and I lost a few kilos, but I found myself still wishing to loose weight. I still didn’t feel at ease with my body, maybe because what I was seeing was still a male body and what I wanted was a female one, a more slender and less muscular one. I decided to keep losing weight so that I could feel more like myself.”

(AMAB)

“At the end, my anorexia was no longer about weight, or about my body shape. I just wanted to die...”

(AMAB)

Compared to restrictive conducts, a desire to gain an excessive amount of weight and bulimic behaviors appeared to be less common in our sample. Looking at AFAB and AMAB groups separately, these behaviors were more common among AFABs (14% of the AFAB group) than among AMABs (6% of the AMAB group). Regarding over-eating behaviors, respondents described having gained significant amounts of weight, especially during puberty. Indeed, these behaviors were described as driven by a self-aware desire to gain weight to hide their body shape and gender marked characteristics. Regarding bulimic behaviors, experiences of purging behaviors (e.g., self-induced vomiting and/or laxative or diuretic misuse)” were described. Concerning the underlying causes of these behaviors, respondents reported being motivated by a desire for self-medication, avoiding weight gain, and altering their body.

“I started to gain weight at the beginning of puberty. Yes, when I was around 13 years old. But then I didn’t stop… I just felt better hiding myself in oversized clothes. I think it was all about concealing my appearance.”

(AFAB)

“When I was 20 or 21, I had about 30 extra pounds, and I couldn’t loose them... I tried following so many diets, I tried doing sports, but nothing worked. It was because somehow, I needed those extra pounds, actually. I only realized it when I started seeing a psychologist and I opened up about how I felt about my gender… it was only then that I managed to loose weight.”

(AMAB)

“There were parts of my body that I just didn’t like... my legs, my hips, my belly... So, I started counting calories and weighing myself, trying to eat in a more healthy way… and then, at some point, I started purging...”

(AFAB)

Respondents also reported compulsive eating behaviors. These conducts were more common among AMABs (20% of the AMAB group) than among AFABs (9% of the AFAB group).

Specifically, respondents reported that their over-eating behaviors mostly occurred between meals, specifying that these conducts initially produced a sense of relief and that they were often connected to feelings of irritability and self-distress. This association is consistent with the literature [46], which underlines that emotion dysregulation is strictly related to binge eating disorders. Indeed, our results highlight that respondents reported more frequent binge episodes when they perceived high levels of distress and uneasiness—for example, on occasions in which they had to show their body in public (e.g., going to the seaside).

“Thinking about food occupies most of my day. Let’s just say that my days can be summed up in being depressed and thinking about food.”

(AMAB)

“When I feel stressed out I tend to eat a lot. Sometimes when I feel very sad and I hate my body, I hate myself, I feel better if I eat. It’s like it soothes the pain.”

(AFAB)

“I always had issues with food, especially during high school. I didn’t eat, I skipped my meals and then I binged at night.”

(AMAB)

### 3.3. Sexuality

Transcript analysis revealed three categories about respondents’ sexual experiences: refusal, discomfort, and risk-taking behaviors (see Table 4).

These results corroborate existing research on gender non-conforming people’s sexuality [47], highlighting the difficulties transgender individuals experience in approaching sexual relations. Indeed, all our study participants reported having experienced or still experiencing feelings of distress and discomfort related to sexuality, even if with different intensity and extension. Specifically, significant difficulties related to sexuality seemed to strongly characterize both AFABs (57%) and AMABs (66%). Most of the participants reported an incapability in engaging in sexual intercourses because of their body dissatisfaction and GI. They described refusing to be touched in any way and/or to show some of their body parts. They also often stated that such avoidance was due to the fact that their body did not correspond to their gender perceived identity.

“I’m a virgin. I reject every kind of physical contact, and it’s not an issue related to the person, it’s not about them being a man or a woman, it’s that I just cannot stand it.”

(AFAB)

Discomfort related to sexual relations also emerged. Inhibition towards physical relationships was reported by most of the respondents They stated they tried to have sexual encounters but that they still perceive severe difficulties with sexuality. Indeed, all respondents underlined a significant lack of pleasant sensations during sexual relations, for example stating that they feel “something missing”.

“I miss something during sex, something of myself. I do not feel right in my body. It’s been a while since I’ve tried to do it–I haven’t had sex in almost two years, because… I mean, I do not feel comfortable doing it, at all.”

(AFAB)

“I usually prefer online sex relationships. The fact that they are online makes me more comfortable. In this way, the other person cannot interact directly with my body. They can only interact with what I am inside, with what I decide to express...”

(AMAB)

On the other hand, some respondents described different coping strategies to handle their discomfort during sexual encounters. For example, some reported having only participated partially in sexual intercourses, remaining dressed, or not letting the other person touch some specific parts of their body associated with a significant feeling of discomfort because of their gender incongruence (e.g., breasts). Others reported using binders or sex toys to deal with their incongruence and an unpleasant feeling of “something missing” during sexual relations.

“I asked my partner if I could remain dressed. I’m glad that everything happened in the dark and that we’ve used a vibrator. I do not know what we would have done otherwise. I do not like sexual penetration, I mean, I really do not like it. I have vaginismus, so it’s just… I really cannot stand it.”

(AFAB)

“I can do other stuff, but I cannot have sex. I cannot even think to have my genitals involved... I cannot even imagine getting an erection. It bothers me too much.”

(AMAB)

Results showed that the discomfort related to sexual relations also affected the respondent’s ability to recognize and communicate their likes and dislikes during sex. Indeed, some participants stated that they consider sex only as a way to please their partners, without being free to express their own desires. This result is consistent with literature that underlines the attempts of transgender individuals to try to have a “different” body, turning their attention to their partners and/or compensating their incongruence between self-image and physical body by making sure their gender “role” during sex is “right” [48]. Thus, body discomfort emerged to be the common aspect of difficulties in sexual relations; sometimes, such discomfort was so severe that respondents referred having troubles even in looking at themselves while they were naked.

“I had a difficult relationship with my body for a lot of time. I had a lot of trouble letting people touch me. Intimacy was too much for me... I hated my body so much that I was not to not being able to shower because I couldn’t see myself naked.”

(AFAB)

Transcript analysis also revealed the presence of risk-taking sexual behaviors such as having a high number of sexual partners, precocious sexual involvement. Abusive sexual experiences and sex work were also reported. Participants described these experiences as related to moments of psychological distress, as an attempt to feel something, or as a way to exert control over their bodies. These results are consistent with studies that underline how psychological well-being and body satisfaction are likely to be associated with positive sexual experiences among transgender individuals [49]. Individuals with body dissatisfaction or body image disorders also often report promiscuous sexual behaviors [50].

“I started having sexual relations at a very early age. I started having sex with older men... I had many doubts about my femininity and having sex with them made me feel like a male. I felt very powerful, even if I couldn’t understand what was happening.”

(AFAB)

“For a lot of time, I had to get drunk to engage in sexual relations. Now I can manage to do it while I’m sober, but I still cannot even look between my legs. There are still parts of me that people cannot touch. If they do, I get embarrassed and leave.”

(AMAB)

## 4. Discussion

The fact that a feeling of incongruence between gender identity and one’s physical appearance may lead to negative outcomes such as self-harming behaviors, eating disorders [7,9] or adverse sexual experiences [48,49] has already been evidenced, but perhaps still not fully understood. Therefore, the present study aimed to further investigate these issues, exploring the personal meaning that individuals with GI give to body dissatisfaction, their description of their own body discomfort, and how their personal characteristics contribute to make body dissatisfaction a risk factor in terms of their overall well-being. Respondents described the different nuances of their body discomfort, underlining that until they did not develop gender marked characteristics, their distress was not perceived as so stressful. This is consistent with the results of some studies [26,51,52] that have highlighted that for individuals with GI the experience of puberty seems associated with high levels of discomfort, since it involves changes that represent a reminder of their gender incongruence. Indeed, a study that focused on puberty changes in transgender adolescents [51] has showed that the use of cross-sex hormones treatment (CSH) alleviates body distress and leads to steady improvements in terms of psychological well-being. Thus, body image and GI exert a strong influence on one another. Indeed, the participants of our study reported that while they were growing up their aversion towards their body increased, and so did their feelings of incongruence. These, in turn, together with the stigma perceived from relationships with peers and the broader social context exposed them to major bullying victimization and to be more prone to social withdrawal. These findings are consistent with the results of several studies [25,53] which have shown that, compared to cisgender youngsters, youths with GI seem to be more exposed to peer rejection, being teased, and social ostracism. Thus, what seems to emerge and needs to be underlined is the significant role that body dissatisfaction has in the severity of GI, which in turn could lead to more negative outcomes regarding psychological distress, and to the development of co-occurring issues. In this regard, respondents reported that their discomfort became so significant that they engaged in self-harm behaviors and suicide attempts in order to feel some relief from it. These results are consistent with the literature [41,54,55] that mentions how individuals with GI are vulnerable to self-harm. More specifically, the presence of a feeling of significant aversion towards one’s body increases the risk of self-injuring conducts and suicidal thoughts. Indeed, respondents described their physical body as the vehicle of their severe suffering; therefore, self-harm behaviors may represent an attempt to control internal conflicts and to contain, at least temporarily, an intolerable sense of disorientation.

Our results also showed that past or current disordered eating conducts such as extreme dieting, binge-eating, and bulimic behaviors are quite common among our sample. These findings are in line with those of several studies that underlined the vulnerability of GI individuals to eating disorders [11,18]. Interestingly, though, the results of our work revealed that GI individuals seem to be quite aware of the underlying causes of these conducts. Respondents indeed reported a high level of self-awareness concerning their body and their attempts to attack and alter it. This is surprising, considering that as literature points out cisgender subjects with eating disorders, usually show a lack of self-awareness regarding their symptoms, or rather that they tend to deny having issues with eating and to “escape” from their negative view of themselves [56]. Specifically, a study conducted by Coutrier and colleagues [57] examining self-awareness in a sample of cisgender individuals with anorexia nervosa (AN) has shown that denial and minimization toward their condition appeared to be common. Alternatively, the high levels of self-awareness among GI individuals could represent an instrumental use of symptomatological categories. As the results show, both AFAB individuals and AMAB individuals showed a tendency to intentionally modify their eating patterns, often in a maladaptive way, in order to alter or “attack” their body. Specifically, AMAB respondents showed restrictive eating patterns, aimed at losing weight and driven by the desire for a more slender and less muscular body—that is, a less masculine one. On the other hand, AFAB respondents showed high levels of binge-eating and bulimic behaviors, reporting feelings of low self-esteem and discomfort as well as a desire to alter their body by gaining weight, in an attempt to modify or neutralize their feminine shape. Such findings highlight how both groups of respondents perceived a desire to alter their body to conform both to their perceived gender and to the cultural and socially constructed ideals of “male” and “female”. In our opinion, this adds a significant facet to our comprehension of gender incongruence and highlights the risk for these individuals to engage in dangerous behaviors. This risk needs to be considered and correctly investigated during the assessment and psychological support of these individuals.

Moreover, respondents also described how their body dissatisfaction created significant difficulties in engaging in sexual relations, to the point that some of them did not consider sexuality a significant part of their lives even though they technically participated in it. They stated that such detachment was related to gender incongruence. Indeed, there are several indications that dissatisfaction with one’s appearance in individuals with GI can make it more difficult to enjoy sexual experiences [48]. Dealing with body discomfort, therefore, is crucial to offer these individuals the possibility of approaching sexuality more positively. Our results also show high rates of risk-taking behaviors. Such behaviors can be interpreted as a way to cope with sexual difficulties, engaging in sexual relations without really becoming intimate with someone, or as a way to have control over an unwanted body.

In summary, our findings show that body discomfort represents the core element of suffering for individuals with GI and a factor of vulnerability for risk-taking and maladaptive behaviors. Thus, a correct assessment of body dissatisfaction in individuals with GI at the first stages of transition processes implies the possibility to better determine treatment eligibility. This, in turn, could prevent the development of maladaptive behaviors and psychological issues.

## 5. Conclusions

Our findings suggest the need for a more thorough understanding of body dissatisfaction in GI individuals. In particular, as previously mentioned, clinicians need to be aware that, in this population, body dissatisfaction can involve negative outcomes that can worsen the suffering of these individuals. Moreover, having addressed how GI individuals suffer from stigmatization, prejudices, and discrimination, a more thorough assessment of their body dissatisfaction may be useful to increase their self-confidence and self-acceptance. For these reasons, future research should investigate more thoroughly body dissatisfaction, assessing it both with standardized instruments [58] and qualitative methods. The latter can indeed offer a better understanding of the personal meaning that GI individuals assign to their body discomfort, therefore allowing clinicians to better comprehend what aspects need to be further investigated and evaluated. Finally, in our opinion, clinicians should enhance supportive interventions focused on encouraging resilience, strength, and self-esteem to help these individuals dealing with their body discomfort and GI.

## 6. Limitations of the Study

Some limitations of the present study should be acknowledged. First, our study was conducted on individuals that have not yet undertaken any intervention for their gender incongruence but were at the early stage of the assessment process. It was fairly predictable that body dissatisfaction among participants could be significantly high. Second, since it is a qualitative study, our results cannot be extended to all the GI population. Moreover, the relatively small size of the sample could have prevented us to get a full picture of the impact of body dissatisfaction in individuals with GI. Finally, cultural and social aspects might also have had a significant role in increasing body dissatisfaction and minority stress in the individuals of the sample, and the study would have benefited from a major analysis of these dimensions. Nevertheless, our results offer a good starting point to better understand how body dissatisfaction could have a key role in increasing suffering among GI individuals.

## Figures and Tables

**Table 1:** Characteristics of sample.

Participants	*N*	Mean	Standard Deviation
AMAB	15	2127	3515
AFAB	21	2100	4037
Total	36	2111	3778

**Table 2:** Domains, categories and subcategories of the area Gender Identity: self-perception.

Domains	Categories	Subcategories	Frequencies
Body dissatisfaction	Discomfort	Feeling ashamed of one’s own body	General
		Repulsion towards the body	General
	Difficult experiences linked to puberty	Body changes	Typical
	Self-Harming/Suicide	Self-harm against the body	Typical
		Suicidal thoughts	Typical
		Suicide attempts	Variant
Lack of self-acceptance	Perception of one’s diversity	Feeling of non-conformity already in childhood	General
	Social relations	Shame	General
		Bullying	Typical
		Social withdrawal	Variant

**Table 3:** Domains, categories and subcategories of the area of Eating Behaviors.

Domains	Categories	Subcategories	Frequencies
Disordered eating patterns	History of disordered eating patterns	Weight monitoring	Variant
		Body-checking	Variant
		Restrictive behaviors	Variant
		Bulimic behaviors	Variant
		Over-eating	Variant
		Compulsive eating behaviors	Variant
	Motivation/Awareness related to eating disorders	Desire for self-care and change of gender	Variant
		Attack to the body	Variant

**Table 4:** Domains, categories and subcategories of the area of Sexuality.

Domains	Categories	Subcategories	Frequencies
Experiences related to sexuality	Refusal	Repulsion towards sexuality	Typical
	Discomfort	Inhibition in physical relationships	Typical
		Inhibition towards body	General
		Coping strategies	Typical
	Risky behaviors	Promiscuous sexuality	Variant
		Trauma/abuses	Variant
